# The RNA-binding protein Hfq assembles into foci-like structures in nitrogen starved *Escherichia coli*

**DOI:** 10.1074/jbc.RA120.014107

**Published:** 2020-06-12

**Authors:** Josh McQuail, Amy Switzer, Lynn Burchell, Sivaramesh Wigneshweraraj

**Affiliations:** Medical Research Council Centre for Molecular Bacteriology and Infection, Imperial College London, London, United Kingdom

**Keywords:** Hfq protein, Escherichia coli (E. coli), photoactivatable localization microscopy (PALM), nitrogen starvation, T7 phage, environmental adaptation, RNA chaperone, gene expression, small regulatory RNA (sRNA), stress response, bacteriophage, gene regulation

## Abstract

The initial adaptive responses to nutrient depletion in bacteria often occur at the level of gene expression. Hfq is an RNA-binding protein present in diverse bacterial lineages that contributes to many different aspects of RNA metabolism during gene expression. Using photoactivated localization microscopy and single-molecule tracking, we demonstrate that Hfq forms a distinct and reversible focus-like structure in *Escherichia coli* specifically experiencing long-term nitrogen starvation. Using the ability of T7 phage to replicate in nitrogen-starved bacteria as a biological probe of *E. coli* cell function during nitrogen starvation, we demonstrate that Hfq foci have a role in the adaptive response of *E. coli* to long-term nitrogen starvation. We further show that Hfq foci formation does not depend on gene expression once nitrogen starvation has set in and occurs indepen-dently of the transcription factor N-regulatory protein C, which activates the initial adaptive response to N starvation in *E. coli*. These results serve as a paradigm to demonstrate that bacterial adaptation to long-term nutrient starvation can be spatiotemporally coordinated and can occur independently of *de novo* gene expression during starvation.

Bacteria in their natural environments seldom encounter conditions that support continuous growth. Hence, many bacteria spend the majority of their time in a state of little or no growth, because they are starved of essential nutrients including carbon, nitrogen, and transitional metals. To maximize chances of survival during prolonged periods of nutrient starvation and facilitate optimal growth resumption when nutrients become replenished, bacteria have evolved complex adaptive strategies. Bacteria initially respond to nutrient deficiency by remodeling gene expression through the synthesis and degradation of RNA. Nitrogen (N) is an essential element of most macromolecules in a bacterial cell, including proteins, nucleic acids, and cell wall components. Thus, unsurprisingly, when *Escherichia coli* cells experience N starvation, they attenuate growth and elicit nitrogen stress response (Ntr response). The Ntr response is activated by the transcription factor Nitrogen regulatory protein C (NtrC) concomitantly with N runout. By directly activating the transcription of *relA*, the gene responsible for the synthesis of the major bacterial stress signaling nucleotide guanosine pentaphosphate, NtrC couples the Ntr response with the stringent response (1) and thereby induces a rapid and large-scale reprograming of gene expression to adjust cellular processes to adapt to N starvation. The Ntr response primarily involves the synthesis of proteins associated with transport and assimilation of nitrogenous compounds into glutamine and glutamate, either catabolically or by reducing the requirement for them in other cellular processes (1–4).

Small regulatory (noncoding) RNA molecules (sRNAs) play an important role in the regulation of gene expression in response to nutrient starvation in many bacteria (5–9). sRNAs can base pair with target mRNAs leading to enhanced translation or inhibition of translation and/or alteration of mRNA stability (10, 11). To form productive interactions with target mRNAs, most sRNAs require an RNA-binding protein. In many bacteria of diverse lineages, the RNA-binding protein Hfq plays a central and integral role in sRNA-mediated regulation of gene expression. Emerging results now reveal that Hfq has diverse gene regulatory functions in bacteria that expand beyond its widely understood role in catalyzing sRNA–mRNA base pairing. These include rRNA processing and assembly of functional ribosomes (12); tRNA maturation (13); and regulation of RNA degradation (14–17). Recently, Hfq was shown to contribute to the distribution of sRNAs to the poles of *E. coli* cells experiencing envelope stress, suggesting a role for Hfq in spatiotemporal regulation of gene expression (18). Although previous studies have demonstrated a role for Hfq in the Ntr response in the N_2_-fixing symbiont *Sinorhizobium meliloti* (19), the role of Hfq-mediated riboregulation in the Ntr response of enterobacteria remains unknown. Hence, the initial aim of this study was to investigate the role of Hfq in regulating the flow of genetic information during the Ntr response *in E. coli*. However, unexpectedly, we uncovered a property of Hfq, which appears to occur independently of changes in gene expression elicited by the NtrC-activated Ntr response, but one that is important for adapting to long-term N starvation.

## Results

### Absence of hfq compromises the ability of E. coli to survive N starvation

**Figure 1.**
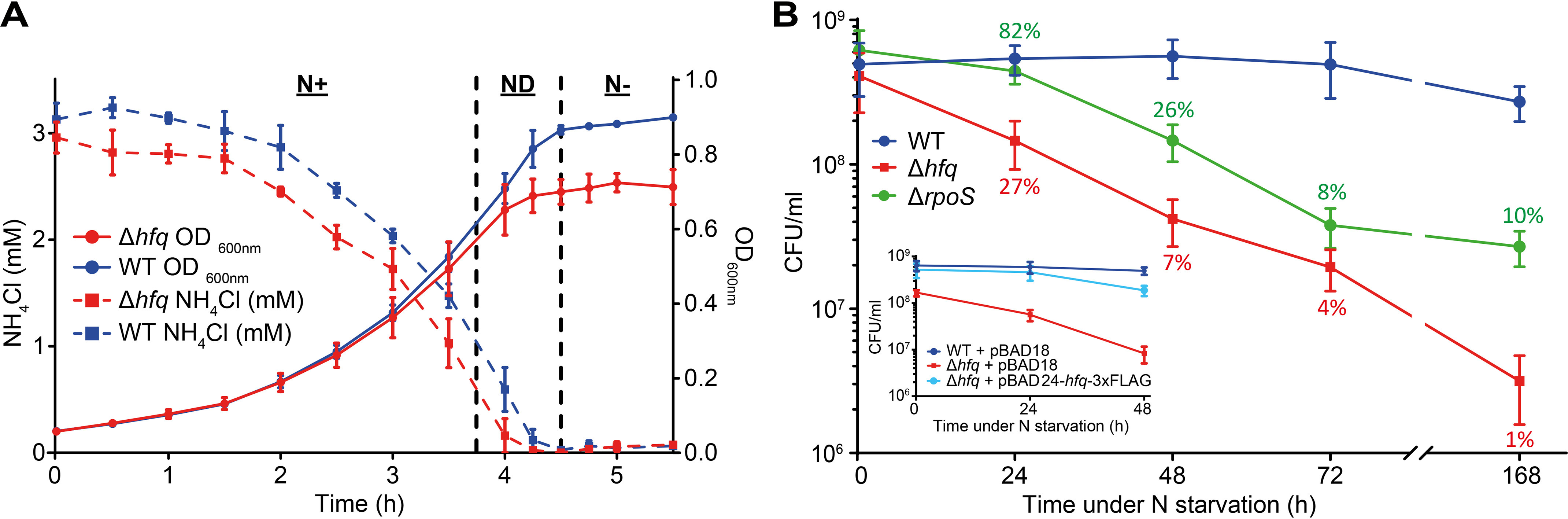
**Absence of *hfq* compromises the ability of *E. coli* to survive N starvation.** *A*, growth and NH_4_Cl consumption of WT and Δ*hfq E. coli* grown in N-limited conditions. *Error bars* represent standard deviation (*n* = 3). *B*, viability of WT, Δ*hfq*, and Δ*rpoS E. coli* during long-term N starvation, measured by counting CFU. The *inset* shows viability of WT and Δ*hfq E. coli* complemented with plasmid-borne *hfq* (pBAD24-*hfq*-3×FLAG) measured by counting CFU. The percentages of Δ*hfq* and Δ*rpoS* viable cells compared that of WT cells are indicated. *Error bars* represent standard deviation (*n* = 3).

To determine whether Hfq has a role in the adaptive response of *E. coli* to N starvation, we grew a batch culture of WT and Δ*hfq E. coli* in a highly defined minimal growth medium with a limiting amount (3 mm) of NH_4_Cl as the sole N source (20). Under these conditions, when NH_4_Cl (*i.e.* N) in the growth medium runs out (N−; Fig. 1*A*), the bacteria enter a state of complete N starvation and subsequent growth attenuation (20). Control experiments revealed that, under our experimental conditions, even 24 h after N had run out (N–24), sufficient glucose still remains available in the growth medium to support growth (12, 10, and 5 mm at N+, N−, and N–24), and consistent with this view, in previous work, we showed that addition of NH_4_Cl to cultures at N− or 24 h after N starvation (N–24) can restart growth (21). As shown in Fig. 1*A*, the initial growth rate (μ) of WT (μ = 64.0 ± 0.30 min/generation) and Δ*hfq* (μ = 67.3 ± 1.28 min/generation) *E. coli* at N+ did not differ greatly. However, as the ammonium chloride levels became gradually depleted (ND; Fig. 1*A*), the growth rate of the Δ*hfq* (μ = 71.3 ± 3.45 min/generation) dropped by ∼8% relative to WT bacteria (μ = 66.1 ± 0.26 min/generation). Both strains attenuated growth at N−, when ammonium chloride levels were depleted (Fig. 1*A*). We then measured the number of colony-forming units (CFU) in the population of WT and Δ*hfq* bacteria as a function of time under N starvation. As shown in Fig. 1*B*, the proportion of viable cells in the WT population moderately increased over the initial 24 h under N starvation but gradually declined as N starvation ensued beyond 24 h. This initial moderate increase in the portion of viable cells over the initial 24 h was not observed for Δ*hfq* bacteria (Fig. 1*B*). In contrast, the portion of viable cells in the Δ*hfq* population rapidly decreased as N starvation ensued. For example, at N–24, only ∼27% of the Δ*hfq* mutant population was viable compared with the WT population (Fig. 1*B*). After 168 h under N starvation (N-168), the majority (∼99%) of bacteria in the Δ*hfq* population were nonviable relative to bacteria in the WT population (Fig. 1*B*). The viability defect of Δ*hfq* bacteria was partially reversible to WT levels when *hfq* was exogenously supplied via a plasmid (Fig. 1*B*, *inset*).

Because Hfq is a pleiotropic regulator of bacterial stress adaptation that largely manifests its effects via functioning as the positive regulator of expression of *rpoS* (22–25), the RNAP promoter-specificity factor (σ^S^), which is responsible for the transcription of diverse stress response–associated genes, we considered whether the inability of Δ*hfq* bacteria to adjust their metabolism to cope with N starvation is due to compromised σ^S^ activity. To investigate this, we calculated, as above, the number of CFUs in the population of Δ*rpoS* bacteria as a function of time under N starvation. The results revealed that, following 24–48 h of N starvation, the Δ*rpoS* bacteria were significantly better at surviving N starvation than Δ*hfq* bacteria (Fig. 1*B*). For example, at N–24, ∼82% of Δ*rpoS* were viable relative to WT bacteria. In contrast, at N–24, only ∼27% of the Δ*hfq* bacteria were viable. After 48 h under N starvation, the Δ*rpoS* bacteria displayed a better ability to survive N starvation than the Δ*hfq* bacteria (Fig. 1*B*). Overall, the results demonstrate that the absence of Hfq compromises the ability of *E. coli* to survive N starvation in a manner that is, to a large extent, independent of Hfq's other roles in bacterial stress adaptation, notably regulation of *rpoS*.

### Absence of hfq compromises the ability of T7 to replicate in long-term N-starved E. coli

**Figure 2.**
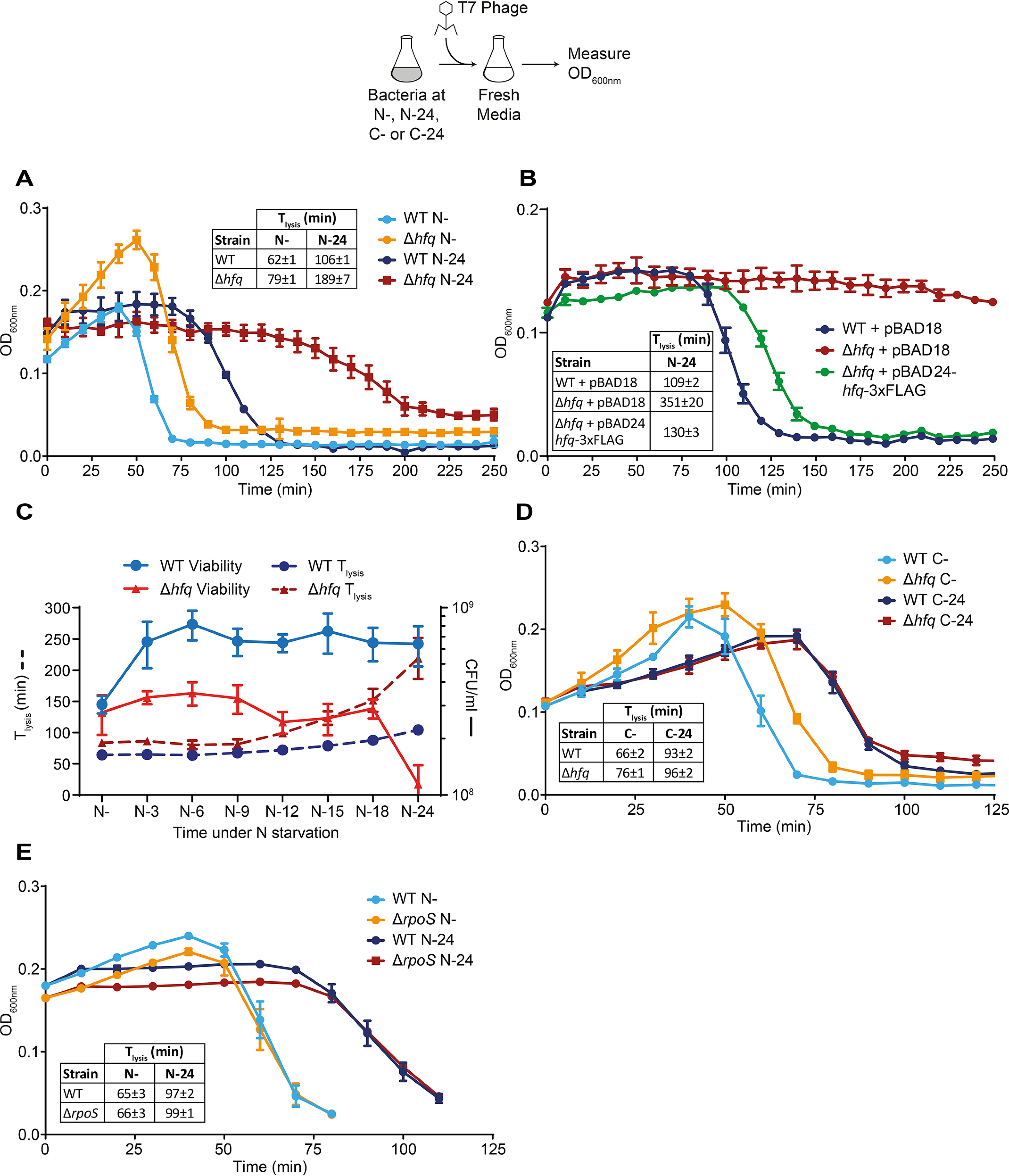
**Absence of *hfq* compromises the ability of T7 to replicate in long-term N-starved *E. coli*.** *A*, graph showing the optical density (*A*_600 nm_) as a function of time of WT and Δ*hfq E. coli* cells from N− and N–24 following infection with T7 phage. The time taken for the starting *A*_600 nm_ value of the culture to decrease by ∼50% (*T*_lysis_) is indicated in the *inset table*. *B*, as in *A*, but the Δ*hfq E. coli* cells were complemented with plasmid-borne *hfq* (pBAD24-*hfq*-3×FLAG). *C*, viability (measured by counting CFU) and *T*_lysis_ of WT and Δ*hfq E. coli* during the first 24 h of N starvation. *D*, as in *A*, but experiments were conducted with carbon-starved bacteria. *E*, as in *A*, but experiments were conducted with Δ*rpoS E. coli* cells. *Error bars* represent standard deviation (*n* = 3).

Because the T7 phage can infect and replicate in exponentially growing and stationary phase (*i.e.* nutrient-starved) *E. coli* cells equally well (26, 27), we used T7 as a biological probe of *E. coli* cell function during N starvation to evaluate the metabolic capacity and capability of the N-starved cellular environment of WT and Δ*hfq* bacteria to support T7 replication. In other words, we expected that, because T7 heavily relies on bacterial resources for replication, any perturbations to these resources, caused by a dysfunctional ability of the Δ*hfq* bacteria to adjust to N starvation, could have a negative impact on the efficacy of T7 replication. Because after 24 h under N starvation there is a substantial decrease in the proportion of viable cells in the Δ*hfq* population (Fig. 1*B*), we initially compared the time it took for T7 to decrease the starting density (*A*_600 nm_) of the culture of WT bacteria at the onset of N starvation (N−) and at N–24 by ∼50% (*T*_lysis_) following infection. We did this by resuspending bacteria from N− and N–24 in medium containing ∼3 mm NH_4_Cl and T7 phage (NH_4_Cl was added to reactivate cellular processes that might be required for T7 infection but might have become repressed upon N starvation). As shown in Fig. 2*A*, the *T*_lysis_ values of cultures of WT bacteria at N− and N–24 were ∼62 and ∼106 min, respectively. The *T*_lysis_ of Δ*hfq* bacteria infected at N− was delayed by ∼17 min compared with WT bacteria, and this resulted in moderate growth of the Δ*hfq* bacterial culture before cell lysis was detectable (Fig. 2*A*). Strikingly, however, T7 replication was substantially compromised in Δ*hfq* bacteria infected at N–24 (Fig. 2*A*), and detectable lysis of Δ*hfq* bacteria at N–24 was delayed by ∼83 min compared with WT bacteria. This delay in lysis of Δ*hfq* bacteria at N–24 was partially reversible to that seen in WT bacteria when *hfq* was exogenously supplied via a plasmid (Fig. 2*B*). Although we are unable to provide an explanation, we note that the Δ*hfq* bacteria at N–24 containing the plasmid without the *hfq* gene (Δ*hfq* + pBAD18 in Fig. 2*B*) displayed a *T*_lysis_ that was ∼162 min longer than Δ*hfq* bacteria without the plasmid (Fig. 2*A*).

It is possible that the increase in *T*_lysis_ seen with Δ*hfq* bacteria at N–24 was simply due to the reduced proportion of viable cells available for T7 to infect and replicate in the mutant population compared with WT population at this time point. To explore this, we measured both viability and *T*_lysis_ as a function time under N starvation over 24 h. The results shown in Fig. 2*C* revealed that although the viability of the Δ*hfq* population remains relatively stable up until N-18, there is a continual increase in *T*_lysis_ after N-9. Hence, we conclude that the observed increase in *T*_lysis_ seen with Δ*hfq* bacteria is not simply due to the decreased proportion of viable cells in the population as N starvation ensues. However, we note that a component of the increase in *T*_lysis_ at N–24 (*i.e.* after N-18) could be due to a combination of the decrease in viable cells in the population, as well as the compromised metabolic capability of the N-starved cellular environment to support efficient T7 replication (caused by the absence of Hfq). Hence, we concluded that the N–24 time point is a suitable and practical (for reasons that will become apparent later) time point to use T7 to probe the metabolic capability of the cellular environment of N-starved *E. coli*.

We next investigated whether the observed difference in *T*_lysis_ between WT and Δ*hfq* bacteria from N–24 was specific to N starvation. Hence, we measured *T*_lysis_ in carbon-starved WT and Δ*hfq* bacteria. This was achieved by growing bacteria in minimal growth medium with a limiting amount of carbon (0.06% (w/v) of glucose instead of 0.4% (w/v) of glucose) and excess NH_4_Cl (10 mm instead of 3 mm). Under these conditions, the bacteria ran out of carbon and thus became carbon-starved before the source of N is completely consumed. As shown in Fig. 2*D*, the dynamics of lysis of the bacterial culture immediately following onset of carbon starvation (C−) and N− were indistinguishable. However, interestingly, experiments with 24-h carbon-starved bacteria (C-24) did not produce a difference in *T*_lysis_ between WT and Δ*hfq* bacteria (Fig. 2*D*) as seen with N–24 bacteria (Fig. 2*A*). Clearly, the compromised ability of T7 to replicate in N–24 bacteria is specific to N starvation. The results also suggest that the compromised ability of T7 to replicate in the Δ*hfq* strain was not simply due to a requirement of T7 for Hfq for replication *per se* and that the ability of T7 to replicate in N-starved *E. coli* is a faithful reporter of *E. coli* cell function during N starvation. Additional experiments with the Δ*rpoS* strain revealed that the compromised ability of T7 to replicate in Δ*hfq* bacteria at N–24 was not due to an indirect effect of Hfq on the regulation of *rpoS* (Fig. 2*E*). This is consis-tent with earlier results showing that the loss of viability of Δ*hfq* bacteria as a function of time under N starvation was not solely due to the loss of *rpoS* activity (Fig. 1*B*). Overall, we conclude that the compromised ability of T7 to replicate in long-term N-starved Δ*hfq* bacteria is predominantly due to a dysfunctional ability of mutant bacteria to adapt as N starvation ensues.

### Hfq molecules assemble into foci-like structures in long-term N-starved E. coli cells

**Figure 3.**
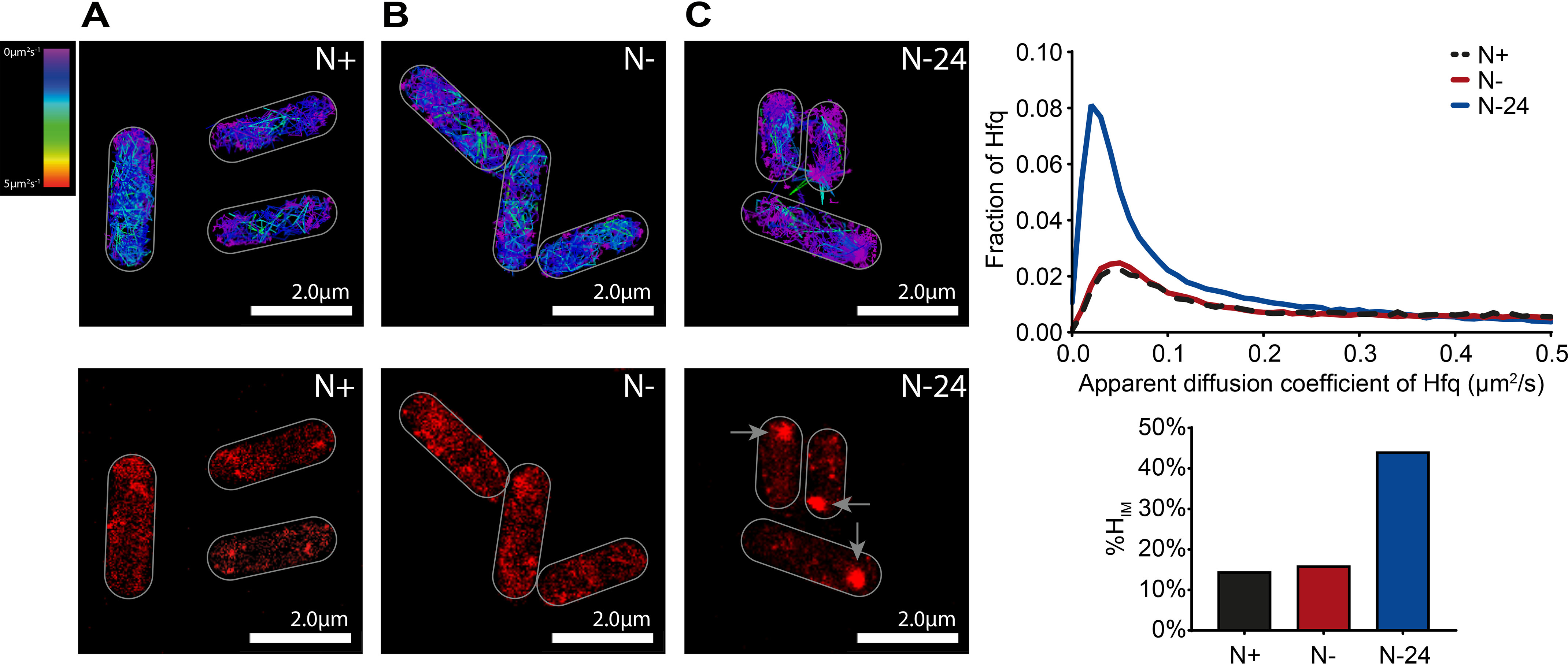
**Hfq molecules assemble into focus-like structures in long-term N-starved *E. coli* cells.** Representative single-molecule tracks (*top panels*) and PALM images (*bottom panels*) of Hfq in *E. coli* cells from N+ (*A*), N− (*B*), and N–24 (*C*) are shown. In the PALM images, the *arrows* indicate the Hfq foci. The *graph* shows the distribution of the apparent diffusion coefficient of Hfq molecules, and the *bar chart* shows %*H*_IM_ values (see text for details).

To explore the molecular basis of the role of Hfq during long-term N starvation in *E. coli*, we used photoactivated localization microscopy combined with single-molecule tracking to study the intracellular behavior of individual Hfq molecules in live *E. coli* cells. To do this, we constructed an *E. coli* strain containing photoactivatable mCherry (PAmCherry) fused C-terminally to *hfq* at its normal chromosomal location. Control experiments established that the ability of (i) PAmCherry-tagged Hfq bacteria to survive N starvation (Fig. S1) and (ii) T7 phage to replicate in 24-h N-starved bacteria containing PAmCherry-tagged Hfq was indistinguishable from that of WT bacteria (Fig. S2). These results thus indicate that the presence of the PAmCherry tag on Hfq did not compromise its function under our experimental conditions. We used the apparent diffusion coefficient (D*) of individual Hfq–PAmCherry molecules, calculated from the mean squared displacement of trajectories, as a metric for the single-molecule behavior of Hfq. As shown in Fig. 3 (*A* and *B*), in bacteria at N+ and N−, the D* values of Hfq molecules were largely similar (although we note that our D* values were ∼6-fold lower than those measured by Persson *et al*. (28) with Dendra2-tagged Hfq in exponentially growing *E. coli* in lysogeny broth, our measurements are largely consistent with those measured by Park *et al*. (29) with mMaple3-tagged Hfq in *E. coli* growing in defined in minimal medium). Of note, the graph in Fig. 3 (and in all subsequent figures) has a truncated *x* axis to emphasize the peaks of interest. In reality, D* values extend to ∼2.0 µm^2^/s for N+ and N− and ∼1.0 µm^2^/s for N–24 (Fig. S3*A*). At N+ and N−, there is a notable portion of Hfq molecules with a diffusion between 0.5 and 2.0 µm^2^/s that is not shown on the graphs in Fig. 3 and all subsequent figures, whereas at N–24 there are very few molecules with a D* of >0.5.

In bacteria from N–24, we detected a large increase in the proportion of molecules with a lower D*. Strikingly, this was due to Hfq forming a *single* focus-like feature (∼360 nm in diameter), which was present usually, but not exclusively, at a cell pole (Fig. 3*C*). These features, hereafter referred to as the Hfq foci, were seen in ∼90% of the cells from N–24 that we analyzed. The D* value of the majority (∼75%) Hfq molecules *within* the Hfq foci was <0.08 (Fig. S3*B*). We collated the D* values of multiple fields of view (typically containing ∼50–300 bacterial cells/field of view) to maximize our data pool. We then used a D* of <0.08 as a threshold to define the relatively immobile population of Hfq molecules within the bacterial cells within the field of view imaged. We then calculated the proportion of all Hfq molecules within this immobile population as a percentage of total number of tracked Hfq molecules within all the bacterial cells within the same field of view imaged to derive a value (%*H*_IM_) to indirectly quantify the efficiency of foci formation by Hfq under different conditions. In other words, cells containing detectable Hfq foci will have an increased %*H*_IM_ compared with cells without detectable Hfq foci and is a value that is derived from typically ∼100–500 bacterial cells. According to this criteria, the %*H*_IM_ values were ∼14, ∼16, and ∼44 in bacteria at N+, N−, and N–24, respectively.

The Hfq foci, once formed, persisted for at least 168 h under N starvation (Fig. S4). We did not detect any foci under identical experimental conditions in N–24 bacteria in *E. coli* strains with PAmCherry fused to RNA polymerase, MetJ (the DNA-binding transcriptional repressor of genes associated with methionine biosynthesis, which is similar in size to Hfq), or ProQ (a new class of sRNA-binding protein in bacteria) (30–32) (Fig. S5). This result suggested that foci formation is a biological property specific to Hfq. Further, we did not detect Hfq foci in bacteria that were starved for carbon for 24 h (Fig. S6). Similarly, we did not detect Hfq foci in 24-h-old stationary phase cultures grown in standard lysogeny broth (Fig. S7). These results suggest that Hfq formation is a phenomenon specific to N starvation. Consistent with this view, we also observed Hfq foci formation in 24-h-old cultures grown in medium containing 3 mm l-glutamine, d-serine, or l-aspartic acid as the sole N source (Fig. S8). Of note, although the %*H*_IM_ values of the Hfq foci in 24-h N-starved bacteria grown in l-glutamine and d-serine were similar to those grown in ammonium chloride, the Hfq foci in 24-h N-starved bacteria grown in l-aspartic acid has a substantially lower %*H*_IM_ value than when grown in ammonium chloride. Nevertheless, these results indicate that Hfq foci formation is a response to depletion of N source and not a response restricted to the depletion of NH_4_Cl. As shown in Fig. S9, the intracellular levels of Hfq did not increase over 24 h under N starvation (when foci have formed). This suggests that the Hfq foci seen in *E. coli* from N–24 are unlikely to be due to accumulation of Hfq as N starvation ensued. To determine whether the foci represent aberrant aggregates of Hfq molecules, we obtained the fraction of aggregated proteins (i.e. in the insoluble fraction) in bacteria from N+, N−, and N–24 (as described in Ref. 33) and attempted to identify Hfq by immunoblotting with anti–mCherry-tag antibodies following separation of the samples on a SDS-polyacrylamide gel. As shown in Fig. S9, we did not detect Hfq in any of the fractions containing aggregated proteins. This suggests that the Hfq foci are unlikely to be aberrant aggregates of Hfq molecules. Fortas *et al*. (34) previously showed that the unstructured C-terminal region of Hfq has the intrinsic property to self-assemble, albeit into long amyloid-like fibrillar structures, *in vitro*. Further, related studies by Taghbalout *et al*. (35) and Fortas *et al*. (34) showed that Hfq forms irregular clusters in exponentially growing *E. coli* cells in lysogeny broth and that the C-terminal region of Hfq was required for this clustering behavior of Hfq, respectively. To investigate whether the C-terminal amino acid residues of Hfq contribute to foci formation, we constructed an *E. coli* strain with a PAmCherry tag fused to *hfq* at its normal chromosomal location that had amino acid residues 73–102 deleted (Hfq_Δ73–102_). As shown in Fig. S10*A*, foci formation by WT Hfq and Hfq_Δ73–102_ did not markedly differ in bacteria at N–24. We note that studies by Vecerek and co-workers (36, 37) showed that a larger truncation of the C-terminal region (Hfq_Δ65–102_) resulted in a mutant protein that was defective in some but not all of its functions. However, consistent with the result that foci formation by Hfq_Δ73–102_ and WT Hfq is indistinguishable, the Hfq_Δ73–102_ variant survived N starvation, as well as WT bacteria (Fig. S10*A*). This suggests that the Hfq foci are different from Hfq clusters/aggregates previously seen *in vitro* and *in vivo*. Finally, Hfq assembles into an hexameric ring-like structure with at least three RNA-binding surfaces located at the proximal face, rim, and distal face of the “ring” (38). To investigate whether foci formation depended on its RNA-binding activity, we measured foci formation by two representative Hfq mutants. The Y25D mutant has compromised mRNA-binding activity (39) and was defective for foci formation compared with WT bacteria (Fig. S10*B*). In contrast, the D9A mutation at the rim of Hfq, which binds RNA with increased affinity but reduced specificity (40), formed foci better than the WT protein (Fig. S10*B*). Thus, it seems that foci formation depends on the RNA-binding activity of Hfq. Overall, we conclude that the majority of Hfq molecules assemble into a single focus-like structure in *E. coli* cells experiencing long-term N starvation.

**Figure 4.**
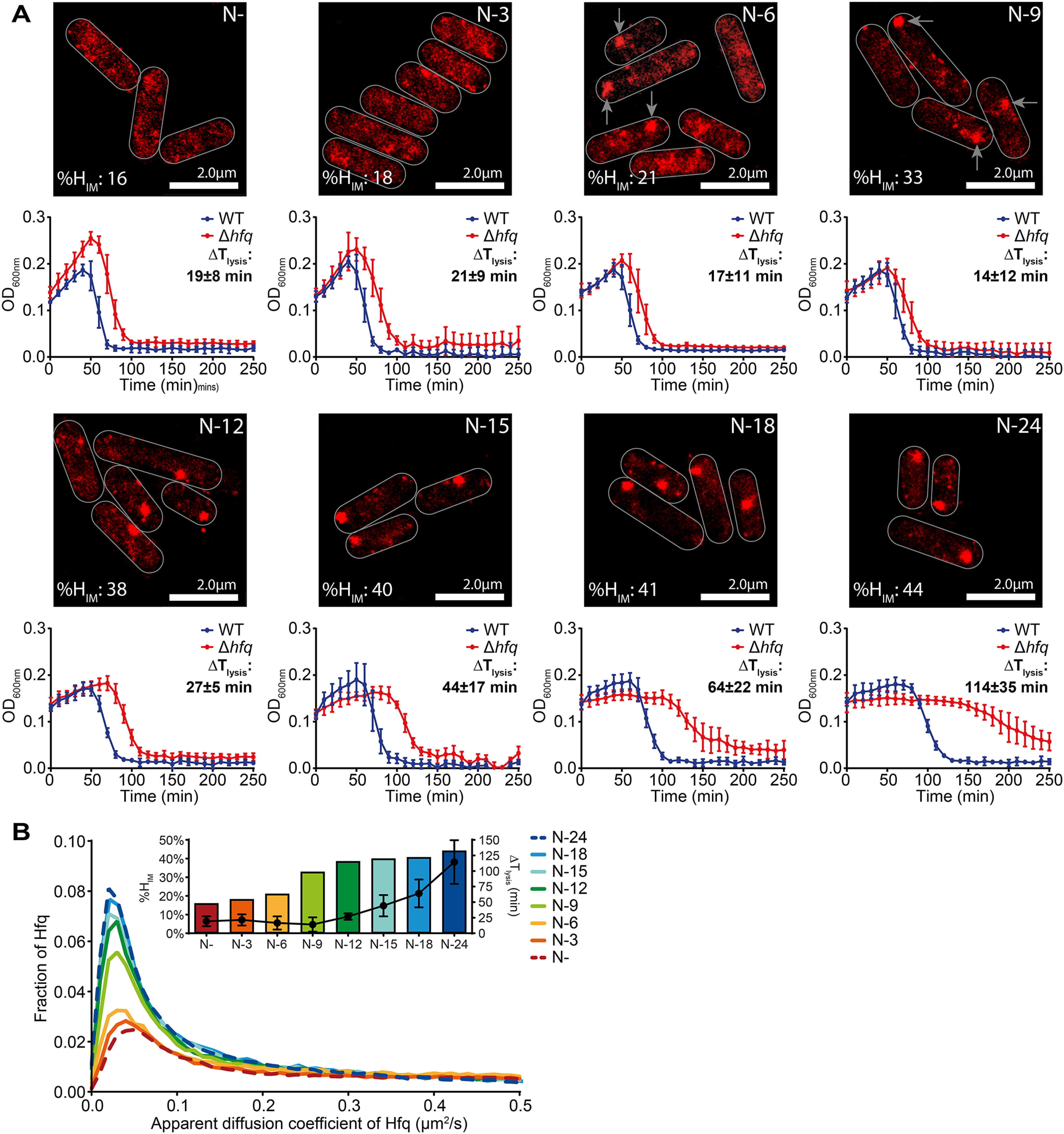
**Hfq foci formation contributes to adapting *E. coli* cell function as N starvation ensues.** *A*, representative PALM images of Hfq in *E. coli* cells as a function of time under N starvation (*top panels*). Images were taken at the indicated time points, and the *arrows* point to the Hfq foci. The images for N− and N–24 have been reused from Fig. 3 (*B* and *C*), respectively, to allow direct comparison. Graphs (*bottom panels*) with the optical density (*A*_600 nm_) as a function of time of WT and Δ*hfq E. coli* cells following infection with T7 phage are shown below each corresponding PALM image. The difference in time taken for the *A*_600 nm_ of the culture to decrease by ∼50% (*T*_lysis_) between WT and Δ*hfq* is indicated (Δ*T*_lysis_). *B*, graphs showing the distribution of apparent diffusion coefficient of Hfq molecules at the different sampling time points and the corresponding %*H*_IM_ and Δ*T*_lysis_ values are shown in the *inset* (see text for details).

**Figure 5.**
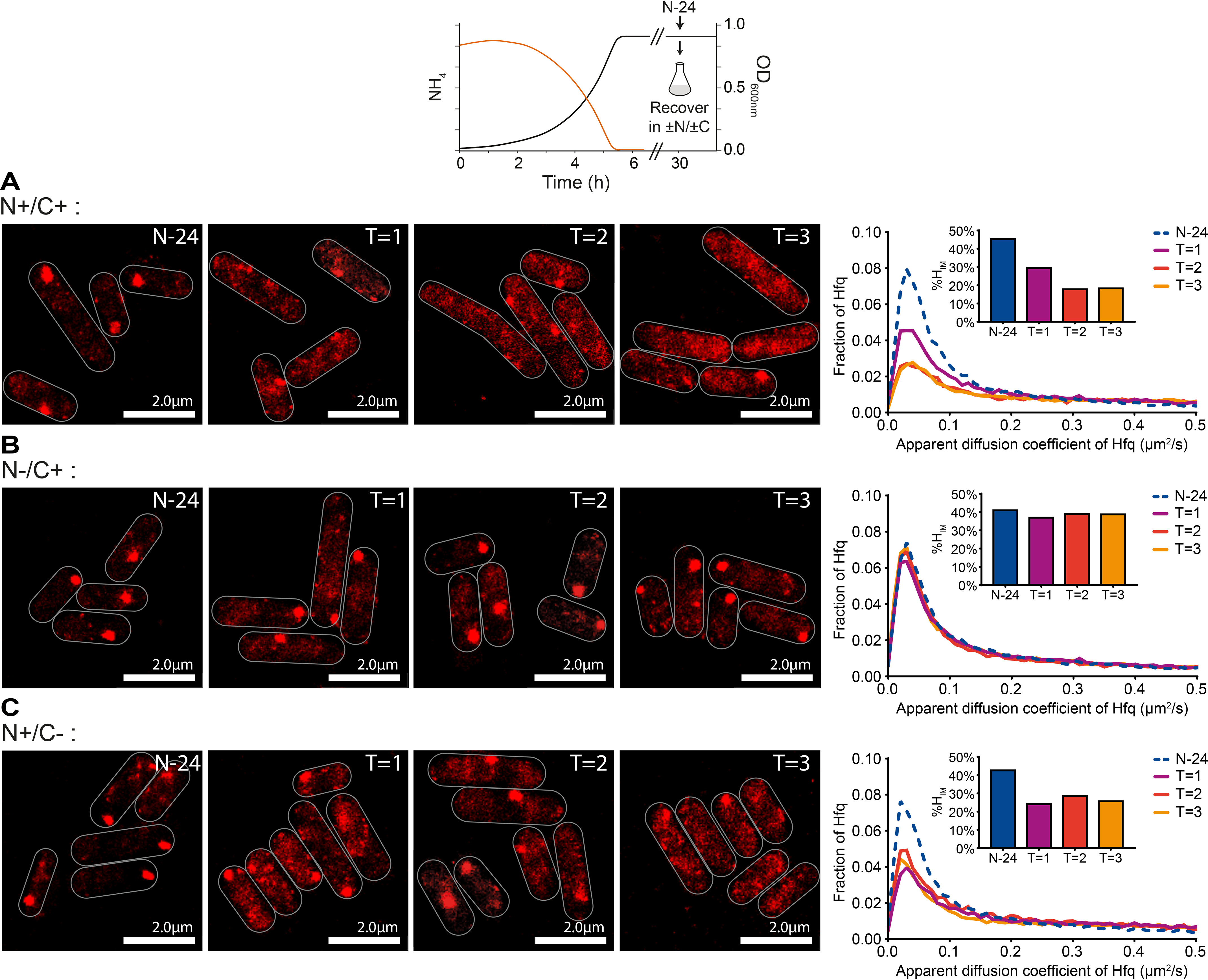
**Hfq foci are reversible.** Shown are representative PALM images of Hfq from N–24 *E. coli* cells resuspended in fresh medium with different combinations of nitrogen and carbon. *A*, N+/C+; *B*, N−/C+; *C*, N+/C−. The images were taken hourly after resuspension. The graphs show the distribution of apparent diffusion coefficient of Hfq molecules at indicated time points with corresponding %*H*_IM_ values shown in *inset graphs*.

**Figure 6.**
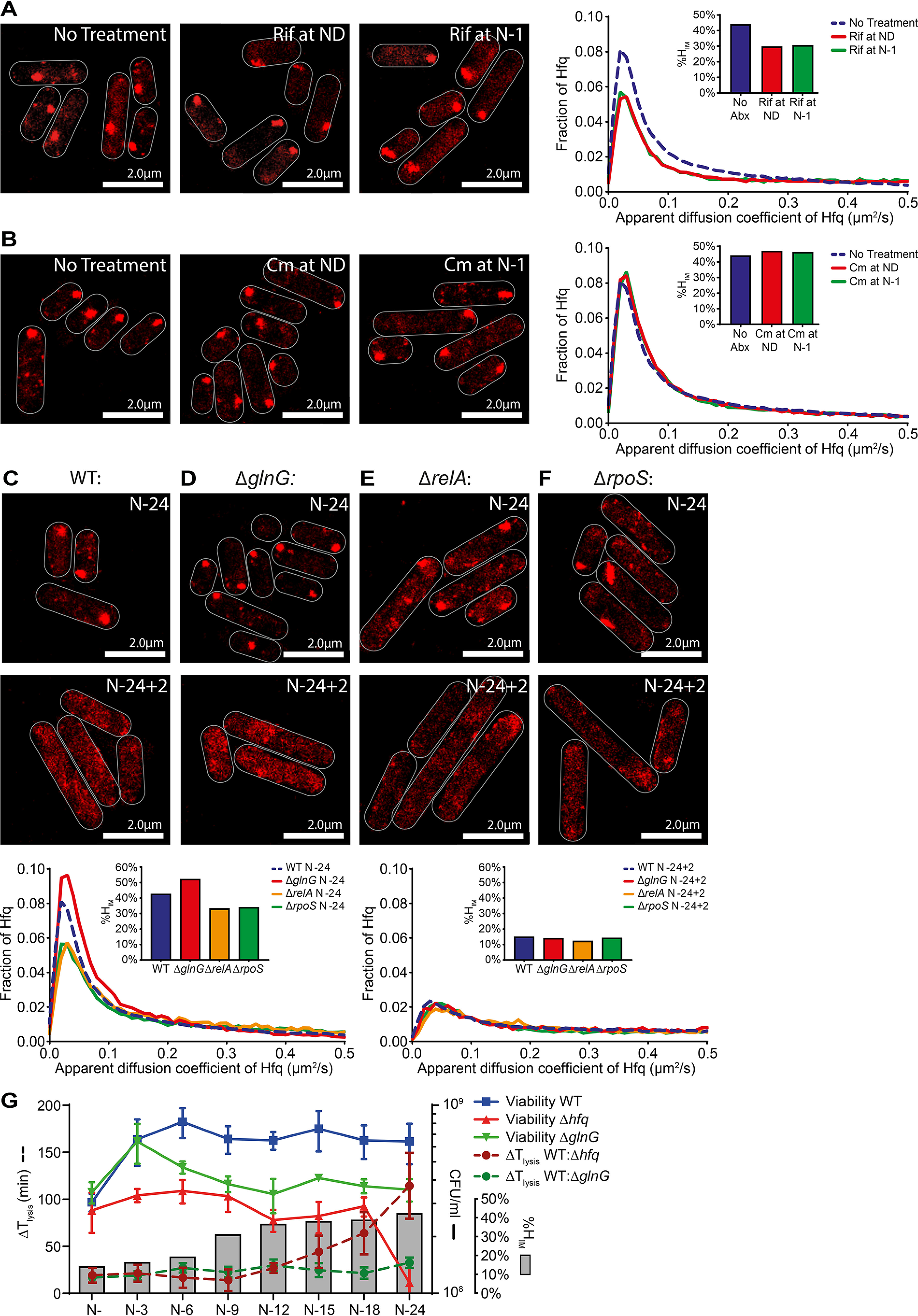
**Hfq foci formation occurs independently of the Ntr response in long-term N-starved *E. coli*.** *A*, representative PALM images of Hfq in *E. coli* cells treated with rifampicin (*Rif*, 100 μg/ml) at ND (*middle panel*) and 1 h following onset of N starvation (N-1) (*right panel*) and imaged at N–24. The untreated bacteria from N–24 are shown for comparison (*left panel*). The *inset graph* shows the distribution of apparent diffusion coefficient of Hfq molecules and corresponding %*H*_IM_ values. *B*, as in *A* but 150 μg/ml of chloramphenicol (*Cm*) was used. *C–F*, representative PALM images of Hfq in *E. coli* cells in WT (*C*), Δ*glnG* (*D*), Δ*relA* (*E*), and Δ*rpoS* (*F*) strains at N–24 and 2 h (N–24 + 2) following alleviation of N starvation stress (as in Fig. 5*A*). The N–24 image for WT has been reused from Fig. 3*C* to allow direct comparison. The graphs show the distribution of apparent diffusion coefficient of Hfq molecules and the %*H*_IM_ values at the sampled time points. *G*, graph showing the viability of WT, Δ*hfq* and Δ*glnG E. coli* during long-term N starvation measured by counting CFU, Δ*T*_lysis_ values of Δ*hfq* and Δ*glnG* relative to WT at each sampling time point, and the corresponding %*H*_im_ values of WT bacteria at each sampling time point.

### Hfq foci formation contributes to adapting E. coli cell function as N starvation ensues

Because the results thus far indicate that Hfq foci formation occurs specifically in long-term N-starved *E. coli*, we next investigated the dynamics of Hfq foci formation during the first 24 h of N starvation. At the same time, we also assessed the ability of T7 to replicate in WT and Δ*hfq* bacteria during the first 24 h of N starvation (by measuring the difference in *T*_lysis_ (Δ*T*_lysis_) between WT and Δ*hfq* bacteria as shown in Fig. 2). We did this to determine whether foci formation by Hfq correlates with the compromised ability of T7 to replicate in Δ*hfq* bacteria (where Hfq foci cannot form). In other words, we reasoned that a dysfunctional adaptive response to N starvation in the Δ*hfq* bacteria (Fig. 2), caused by the inability to form the Hfq foci, would render the cellular environment inadequate for efficient T7 replication. As shown in Fig. 4*A*, no Hfq foci were detected in bacteria that had been N-starved for up to 3 h (*i.e.* at N-3). However, in bacteria that have been starved of N for ∼6 h (N-6), we began to detect clustering of Hfq resembling Hfq foci and by N-12 discernible Hfq foci were clearly seen (Fig. 4*A*). Strikingly, a substantial increase in Δ*T*_lysis_ in Δ*hfq* bacteria occurred concomitantly with the assembly of Hfq into the foci in WT bacteria (Fig. 4, *A* and *B*, compare N-9 with N-12, N-15, N-18, and N–24). However, we note that this concomitant increase in Δ*T*_lysis_ in Δ*hfq* bacteria and timing of foci formation in WT bacteria do not perfectly correlate (Fig. 4*A*, compare N-9, N-12, N-15, and N-18). Because the adaptive processes to N starvation are to ensure that metabolic processes are adjusted to maximize chances of survival, any perturbation to this adaptive process is unlikely to result in an instantaneous observable phenotype. Hence, we suggest that the phenotypic consequences (*i.e.* T7 replication in this case) of perturbing the adaptive response to N starvation by disrupting foci formation (as in the Δ*hfq* bacteria) is only perceived after a delay. We conclude that Hfq foci formation is a process that occurs gradually over the course of N starvation in *E. coli* and that Hfq foci have a specific role in adapting *E. coli* cell function as N starvation ensues. In further support of this conclusion, we observe neither assembly of Hfq into foci in long-term carbon-starved WT bacteria (Fig. S6) nor a defect in the ability of T7 to replicate in carbon-starved Δ*hfq* bacteria (Fig. 2*D*).

### Hfq foci are reversible

We next considered that if Hfq foci formation is a direct response to long-term N starvation, then the foci should dissipate when long-term N-starved bacteria are replenished with N. To explore this, we harvested N-starved (hence growth-attenuated) bacteria at N–24 and inoculated them into fresh growth medium. This, as expected, resulted in the resumption of growth and we detected the dissipation of the Hfq foci just ∼1 h after inoculation in fresh N replete growth medium (Fig. 5*A*). To establish whether the dispersion of the Hfq foci was a direct response to the presence of N or because of resumption of growth, we repeated the experiment and inoculated bacteria from N–24 into fresh growth medium that was devoid of either nitrogen or carbon, which could not support the resumption of growth. In medium devoid of N (but that contained carbon), we failed to detect the dissipation of the Hfq foci even after ∼3 h after inoculation (Fig. 5*B*). However, strikingly, the Hfq foci begun to dissipate upon inoculation into medium that only contained N but not carbon (Fig. 5*C*). Thus, we conclude that the Hfq foci are reversible and that their formation and dissipation are a direct response to the availability of N. Further, the reversibility of the Hfq foci also serves to alleviate concerns that the formation of the Hfq foci is due to the inherent tendency of some mCherry-tagged proteins to aberrantly aggregate (41).

### Hfq foci formation is an element of the Ntr response that occurs independent of NtrC and de novo gene expression in long-term N-starved E. coli

The results thus far suggest that foci formation by Hfq is involved in adapting to long-time N starvation. We next wanted to find out whether Hfq foci formation was part of the NtrC-activated Ntr response, which is the initial major re-sponse to N deficiency. Because the NtrC-activated Ntr response primarily manifests in global changes in gene expression in response to N starvation, we investigated whether perturbing the transcriptional and translational response with the RNA polymerase inhibitor rifampicin and 50S ribosomal protein inhibitor chloramphenicol, respectively, affected Hfq foci formation. As shown in Fig. 6 (*A* and *B*), the addition of 100 μg/ml rifampicin or 150 μg/ml chloramphenicol to bacteria prior to N− (ND; Fig. 1*A*) or 1 h after N− (N-1) did not prevent Hfq foci formation at N–24 compared with untreated cells. In the case of rifampicin-treated cells, we note that the %*H*_IM_ value is lower than in untreated cells (%*H*_IM_ = ∼30 treated; %*H*_IM_ = ∼44 untreated). We suggest that this is could be due to the effects of the decompaction of the nucleoid, caused by rifampicin treatment, on the mobile population of Hfq molecules, as a less condensed nucleoid tends to facilitate higher diffusibility of mobile proteins in the cell (42). The observation that Hfq foci still form 24 h following N starvation, when transcription or translation is perturbed at ND, suggests that Hfq foci formation occurs independently of the NtrC-activated Ntr response. Consistent with this view, the observation that Hfq foci still form 24 h following N starvation when transcription or translation is perturbed at N-1, *i.e.* once N has run out and bacterial growth has attenuated as a consequence, suggests that Hfq foci formation does not depend on *de novo* transcription or translation once N starvation has set in.

Based on these two observations, we expected that Hfq foci formation would be unaffected when the Ntr response is specifically perturbed. Hence, we measured the dynamics of Hfq foci formation in mutant bacteria devoid of NtrC (Δ*glnG*), the master transcriptional regulator of the classically characterised Ntr response. As previously reported, the absence of NtrC does not prevent *E. coli* from growing under our experimental conditions, although mutant bacteria grew with a moderately slower doubling time than WT bacteria (1). As shown in Fig. 6*D*, Hfq foci were detected in Δ*glnG* bacteria in response to N starvation at N–24, although the Hfq foci appeared to form moderately faster in the mutant bacteria compared with WT bacteria and resulted in mutant bacteria having a slightly increased %*H*_IM_ value of 52 compared with WT bacteria that has a %*H*_IM_ of 44 (Fig. 6*C*; also see Fig. S11). Further, the absence of NtrC did not affect the ability of the Hfq foci to dissipate when the N starvation stress was alleviated (Fig. 6*D*). It seems that Hfq foci formation occurs independently of the NtrC-activated Ntr response and the gene expression changes that accompany this response. Consistent with this view, as shown in Fig. 6 (*E* and *F*) and Fig. S11, the absence of downstream effectors of the NtrC-activated Ntr response, RelA or RpoS, also did not prevent Hfq foci formation at N–24, although foci formation in Δ*relA* and Δ*rpoS* bacteria occurred moderately slower compared with WT bacteria and resulted in mutant bacteria having a slightly reduced %*H*_IM_ value compared with WT bacteria (Fig. 6, *E* and *F*, and Fig. S11). As in the Δ*glnG* bacteria, the Hfq foci dissipated when N–24 Δ*relA* and Δ*rpoS* bacteria were replenished with N (Fig. 6, *E* and *F*). Further, we note that the Hfq foci in some Δ*relA* and Δ*rpoS* bacterial cells at N–24 were either absent or irregularly shaped compared with WT bacteria (compare Fig. 6*C* with Fig. 6, *E* and *F*; also see Fig. S11). It is thus possible that the inability to synthesize the signaling nucleotide guanosine pentaphosphate (p)ppGpp (Δ*relA*) and the pleiotropic downstream effects of (p)ppGpp (*e.g.* on *rpoS* levels) could partially impair the Hfq foci.

Because the results thus far indicate that foci formation by Hfq is not a constituent of the NtrC-activated Ntr response to N starvation, we next compared how the absence of NtrC affected the viability of *E. coli* and the ability of T7 to replicate during long-term N starvation. As shown in Fig. 6*G*, the proportion of viable cells in the population of Δ*glnG* bacteria decreased, on average, by ∼50% after 24 h under N starvation; although the loss of viability was substantially more pronounced in the population of Δ*hfq* bacteria (Figs. 1*A* and 6*G*). Intriguingly, the Δ*T*_lysis_ values for the Δ*glnG* bacteria did not markedly differ as N starvation ensued over 24 h. In contrast, as indicated above, this was not the case for the Δ*hfq* bacteria, and we observed an increase in Δ*T*_lysis_ after when the Hfq foci would normally become established (Figs. 4 and 6*G*). Overall, we conclude that Hfq foci formation is a response to long-term N starvation that occurs independently of the NtrC-activated Ntr response and *de novo* gene expression once N starvation has set in.

## Discussion

Although the adaptive response to N starvation, the Ntr response, in *E. coli* has been well-investigated, many of these studies have been conducted with bacteria subjected to short-term N starvation (4, 20, 43–45). This study has uncovered an aspect of the Ntr response in *E. coli*, which involves the RNA-binding protein Hfq, that is temporally coordinated and does not depend on *de novo* gene expression. This study has unraveled that, as N starvation ensues, Hfq forms a single and reversible focus-like structure in long-term N-starved *E. coli* cells. These Hfq foci seen in long-term N-starved *E. coli* cells are markedly distinct from clustering of Hfq molecules seen previously in *E. coli* under different growth or stress conditions (18, 34, 35). Significantly, the formation of the Hfq foci occurs independently of (i) NtrC, which activates the classically characterized Ntr response at the onset of N starvation, and (ii) (the accompanying) *de novo* gene expression in bacteria experiencing N starvation. Although the identity of the signal that induces Hfq foci formation remains elusive, the observation that the Hfq foci form more rapidly in the Δ*glnG* bacteria (Fig. S11) is consistent with the idea that if bacteria cannot adapt initially via the Ntr system, the stress signal to allow foci formation may accumulate sooner during N starvation. Intriguingly, this signal seems to occur independent of *de novo* gene expression. It is hence tempting to speculate that the stress signal that induces Hfq foci is depletion of a protein and/or nucleic acid and/or the concomitant accumulation of a decay product. Because Hfq is a global regulator of bacterial cell function, it is difficult to unambiguously separate, at this stage in our analyses, the other functions of Hfq from its specific role in the Ntr response in mechanistic detail. Although future work will now focus on defining the composition and organization of the Hfq foci, the experiments with T7 indicate that the Hfq foci contribute to adapting *E. coli* cell function as N starvation ensues. We speculate that the Hfq foci could be a ribonucleoprotein complex that de-grades unwanted RNA molecules or ribosomes to release N from the nitrogenous RNA molecules or sequester cellular resources to maximize the chances of survival as N starvation conditions ensue. Although our results indicate that Hfq foci formation depends on its RNA-binding activity (Fig. S10*B*), attempts to specifically stain RNA (using commercially available RNA specific stains) in N–24 bacteria, when Hfq foci have formed, proved to be unsuccessful. We suspect this is due to the extremely poor permeability of the bacteria at N–24. We suggest that the absence of Hfq foci could result in the “mismanagement” of cellular processes as N starvation ensues. Such a scenario would explain the inability of T7 phage, which heavily relies on host bacterial resources, to replicate in N–24 Δ*hfq* bacteria, in which the Hfq foci cannot form. It is thus tempting to speculate that the Hfq foci resemble ribonucleoprotein complexes observed in stressed eukaryotic cells, which form by liquid–liquid phase separation and are involved in broad aspects of managing RNA metabolism as direct response to stress (46–48). In fact, stress-response–associated liquid–liquid phase separated ribonucleoprotein complexes, called BR-bodies, have been recently observed in the α-proteobacterium *Caulobacter crescentus* (49, 50). Indeed, the gradual accumulation of the Hfq foci as a function of time under N stress, their size, and their dissipation upon alleviation of the N stress, are some of the properties the Hfq foci share with ribonucleoprotein complexes observed in stressed eukaryotic cells.

In summary, a recent study by Kannaiah *et al*. (18) and this study have now independently shown that Hfq has the propensity to assemble into foci-like structures in *E. coli* experiencing stress. In case of the former study, the Hfq foci formed to locate small RNAs at the poles of the cells as a mechanism of spatiotemporal regulation of gene expression in response to envelope stress. The spatiotemporal regulation of bacterial processes is an emerging area of research in bacteriology, and the Hfq foci described in this study represents a mechanism to spatiotemporally manage cell function during long-term N starvation. Importantly, this study has also indicated that adaptive processes in nutrient-starved and growth-attenuated bacteria can occur independently of *de novo* gene expression and major regulatory factors that have evolved to allow bacteria adapt to nutrient stresses.

## Materials and methods

### Bacterial strains and plasmids

All strains used in this study were derived from *E. coli* K-12 and are listed in Table S1. The Hfq–PAmCherry and MetJ-PAmCherry strains were constructed using the λ Red recombination method (51) to create an in-frame fusion encoding a linker sequence and PAmCherry, followed by a kanamycin resistance cassette (amplified from the KF26 strain (42)) to the 3′ end of *hfq* and *metJ*. The ProQ-PAmCherry reporter strain (Δ*proQ*+pACYC*-proQ-PAmCherry*) was constructed using a Δ*proQ* strain (Provided by Prof. Jörg Vogel, University of Würzburg). The pACYC-ProQ-PAmCherry plasmid was made by Gibson assembly and used to express ProQ-PAmCherry under the native promoter of *proQ* (52). The Hfq_Δ73–102_–PAmCherry strain was made using the λ Red recombination method (51), similar to construction of the Hfq–PAmCherry strain, but with an in-frame fusion of the linker–PAmCherry sequence to replace amino acids 73–102. Gene deletions (Δ*glnG*, Δ*relA*, or Δ*rpoS*) were introduced into the Hfq–PAmCherry strain as described previously (20). Briefly, the knockout alleles were transduced using the P1*vir* bacteriophage with strains from the Keio collection (53) serving as donors.

### Bacterial growth conditions

Bacteria were grown in Gutnick minimal medium (33.8 mm KH_2_PO_4_, 77.5 mm K_2_HPO_4_, 5.74 mm K_2_SO_4_, 0.41 mm MgSO_4_) supplemented with Ho-LE trace elements (54), 0.4% (w/v) glucose as the sole carbon source and NH_4_Cl as the sole N source. Overnight cultures were grown at 37 °C, 180 rpm in Gutnick minimal medium containing 10 mm NH_4_Cl. For the N starvation experiments, 3 mm NH_4_Cl was used (see text for details). For carbon starvation experiments, bacteria were grown in Gutnick minimal medium containing 22 mm glucose and 10 mm NH_4_Cl. The NH_4_Cl and glucose concentrations in the media were determined using the Aquaquant ammonium quantification kit and glucose assay kit, respectively (both by Merck Millipore), according to the manufacturer's instructions. The proportion of viable cells in the bacterial population was determined by measuring CFU/ml from serial dilutions on lysogeny broth agar plates. Complementation experiments used pBAD24-*hfq*-3×FLAG or pBAD18 as the empty vector control, in Gutnick minimal medium supplemented with 0.2% (w/v) l-arabinose at *t* = 0, for induction of gene expression. To observe Hfq foci dissipation, 25 ml of N–24 culture was centrifuged at 3,200 *g* and resuspend in fresh Gutnick minimal medium containing different combinations of 0.4% (w/v) glucose and 3 mm NH_4_Cl (see text for details).

### Photoactivated localization microscopy (PALM) and single-molecule tracking (SMT)

For the PALM and SMT experiments, the Hfq–PAmCherry (and derivatives), KF26, MetJ-PAmCherry, and ProQ-PAmCherry reporter strains were used. The bacterial cultures were grown as described above, and samples were taken at the indicated time points, then imaged, and analyzed as previously described (42, 55). Briefly, 1 ml of culture was centrifuged, washed, and resuspended in a small amount of Gutnick minimal medium without any NH_4_Cl + 0.4% glucose; samples taken at N+ were resuspended in Gutnick minimal medium with 3 mm NH_4_Cl + 0.4% glucose. For the carbon starvation experiments, C− and C-24 samples were resuspended in Gutnick minimal medium with 10 mm NH_4_Cl but no glucose; C+ samples were resuspended in Gutnick minimal medium with 10 mm NH_4_Cl and 0.06% glucose. 1 μl of the resuspended culture was then placed on a Gutnick minimal medium agarose pad (1× Gutnick minimal medium with no NH_4_Cl + 0.4% glucose with 1% (w/v) agarose); samples taken at N+ were placed on a pad made with Gutnick minimal medium with 3 mm NH_4_Cl. For the carbon starvation experiments, C− and C-24 samples were placed on a pad containing 10 mm NH_4_Cl but no glucose; C+ samples were placed on a pad containing 10 mm NH_4_Cl and 0.06% glucose. The cells were imaged on a PALM-optimized Nanoimager (Oxford Nanoimaging) with 15-μs exposures at 66 frames/s over 10,000 frames. Photoactivatable molecules were activated using 405- and 561-nm lasers. For SMT, the Nanoimager software was used to localize the molecules by fitting detectable spots of high photon intensity to a Gaussian function. The Nanoimager software SMT function was then used to track individual molecules and draw trajectories of individual molecules over multiple frames, using a maximum step distance between frames of 0.6 μm and a nearest-neighbor exclusion radius of 0.9 μm. The software then calculated the apparent diffusion coefficients (D*) for each trajectory over at least four steps, based on the mean squared displacement of the molecule.

### Immunoblotting

Immunoblotting was conducted in accordance to standard laboratory protocols (56). The following antibodies were used: rabbit polyclonal anti-mCherry at 1:1,000 dilution (abcam, AB167453) and anti-rabbit IgG HRP (BioLegend, NA934-1ML) at 1:10,000 dilution. ECL Prime Western blotting detection reagent (GE Healthcare, RPN2232) was used to develop the blots, which were analyzed on the ChemiDoc MP imaging system, and bands were quantified using Image laboratory Software.

Insoluble protein fractions were purified exactly as previously described (33). Briefly, aliquots of bacterial cultures (5–20 ml) were cooled on ice, and cell pellets were harvested by centrifugation at 3,200 *g* for 10 min at 4 °C. To extract the insoluble protein fraction, the pellets were resuspended in 40 μl of buffer A (10 mm potassium phosphate buffer, pH 6.5, 1 mm EDTA, 20% (w/v) sucrose, 1 mg/ml lysozyme) and incubated on ice for 30 min. The cells were lysed by adding 360 μl of buffer B (10 mm potassium phosphate buffer, pH 6.5, 1 mm EDTA) followed by sonication (10-s pulse on, 10-s off, 40% amplitude, repeated six times (2 min of total time)) on ice. Intact cells were removed by centrifugation at 2,000 *g* for 15 min at 4 °C. The insoluble fraction was collected by centrifugation at 15,000 *g* for 20 min at 4 °C and kept at −80 °C. Pellets were resuspended in 400 μl of buffer B and centrifuged at 15,000 *g* for 20 min at 4 °C. The pellet was then resuspended in 320 μl of buffer B plus 80 μl of 10% (v/v) Nonidet P-40. Aggregated proteins were then collected by centrifugation at 15,000 *g* for 30 min at 4 °C. This wash step was repeated. The Nonidet P-40 insoluble pellets were washed with 400 μl of buffer B. The final insoluble protein pellet was resuspended in 200 μl of buffer B and 200 μl of LDS loading dye, and samples were run on a 12% (w/v) SDS-polyacrylamide gel and analyzed by Coomassie stain and immunoblotting, in accordance with standard laboratory protocols (56, 57). To obtain whole cell extracts, bacterial pellets were resuspended in 40 µl of buffer A (with no lysozyme), and 50 µl of 2 × lithium dodecyl sulfate loading dye was added before boiling the sample for 5 min and analysis using SDS-polyacrylamide gel separation as above.

### T7 phage infection assay

Bacterial cultures were grown in Gutnick minimal medium as described above to the indicated time points. The samples were taken and diluted to *A*_600 nm_ of 0.3 in Gutnick minimal medium containing ∼3 mm NH_4_Cl to a final volume of 500 μl and transferred to a flat-bottomed 48-well plate, together with T7 phage at a final concentration of 4.2 × 10^9^ phage/ml. The cultures were then grown at 37 °C with shaking at 700 rpm in a SPECTROstar Nano microplate reader (BMG LABTECH), and *A*_600 nm_ readings were taken every 10 min.

## Data availability

Data will be shared upon request to the corresponding author, Sivaramesh Wigneshweraraj.

## Supplementary Material

Supporting Information
