# Supplementary material for: The RNA-binding protein Hfq assembles into foci-like structures in nitrogen starved Escherichia coli
Source: J Biol Chem. 2020 Jun 12;295(35):12355–67. doi: 10.1074/jbc.RA120.014107 (PMC7458820; doi:10.1074/jbc.RA120.014107)
Supplement: Supporting Information [file supp_295_35_12355__index.html]

The RNA-binding protein Hfq assembles into foci-like structures in nitrogen starved Escherichia coli — Formation of Hfq foci during nitrogen starvation — Supporting Information 

# The RNA-binding protein Hfq assembles into foci-like structures in nitrogen starved *Escherichia coli*

## Supporting Information

- Supporting Information (to be published online) - Supplementary information
