# Supplementary material for: The RNA-binding protein Hfq assembles into foci-like structures in nitrogen starved Escherichia coli
Source: J Biol Chem. 2020 Jun 12;295(35):12355–67. doi: 10.1074/jbc.RA120.014107 (PMC7458820; doi:10.1074/jbc.RA120.014107)
Supplement: Supporting Information [file supp_RA120.014107_160474_2_supp_546750_qbrv9w.pdf]

The RNA-binding protein Hfq assembles into foci-like structures in nitrogen starved Escherichia coli

Supporting Information

Josh McQuail, Amy Switzer, Lynn Burchell and Sivaramesh Wigneshweraraj*

MRC Centre for Molecular Bacteriology and Infection, Imperial College London, London, SW7 2AZ, UK

Figure S1

Figure S2

Figure S3

Figure S4

Figure S5

Figure S6

Figure S7

Figure S8

Figure S9

Figure S10

Figure S11

Table S1

Figure S1

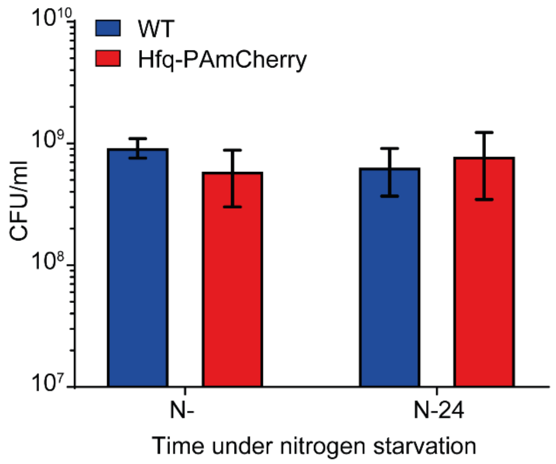

Fig. S1. The PAmCherry tag on Hfq does not affect cell viability. Viability of WT and Hfq-PAmCherry *E. coli* at N- and N-24 measured by counting CFU. Error bars represent standard deviation (n = 3).

Figure S2

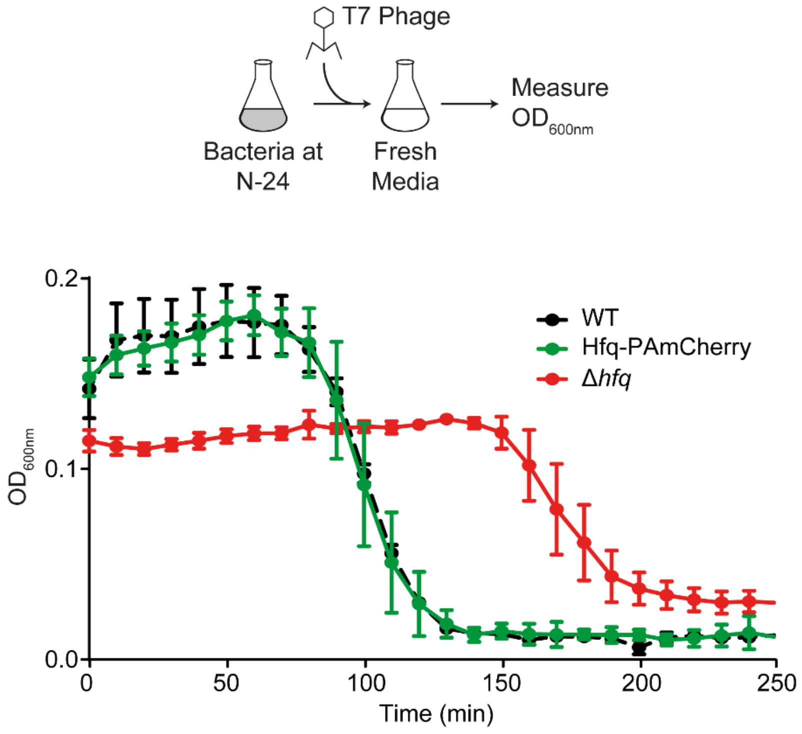

Fig. S2. The PAmCherry tag on Hfq does not affect the ability for T7 phage to replicate *E. coli* cells. Graph showing the optical density as a function of time of WT, Hfq-PAmCherry and Δ*hfq E. coli* cells from N-24 following infection with T7 phage.

Figure S3

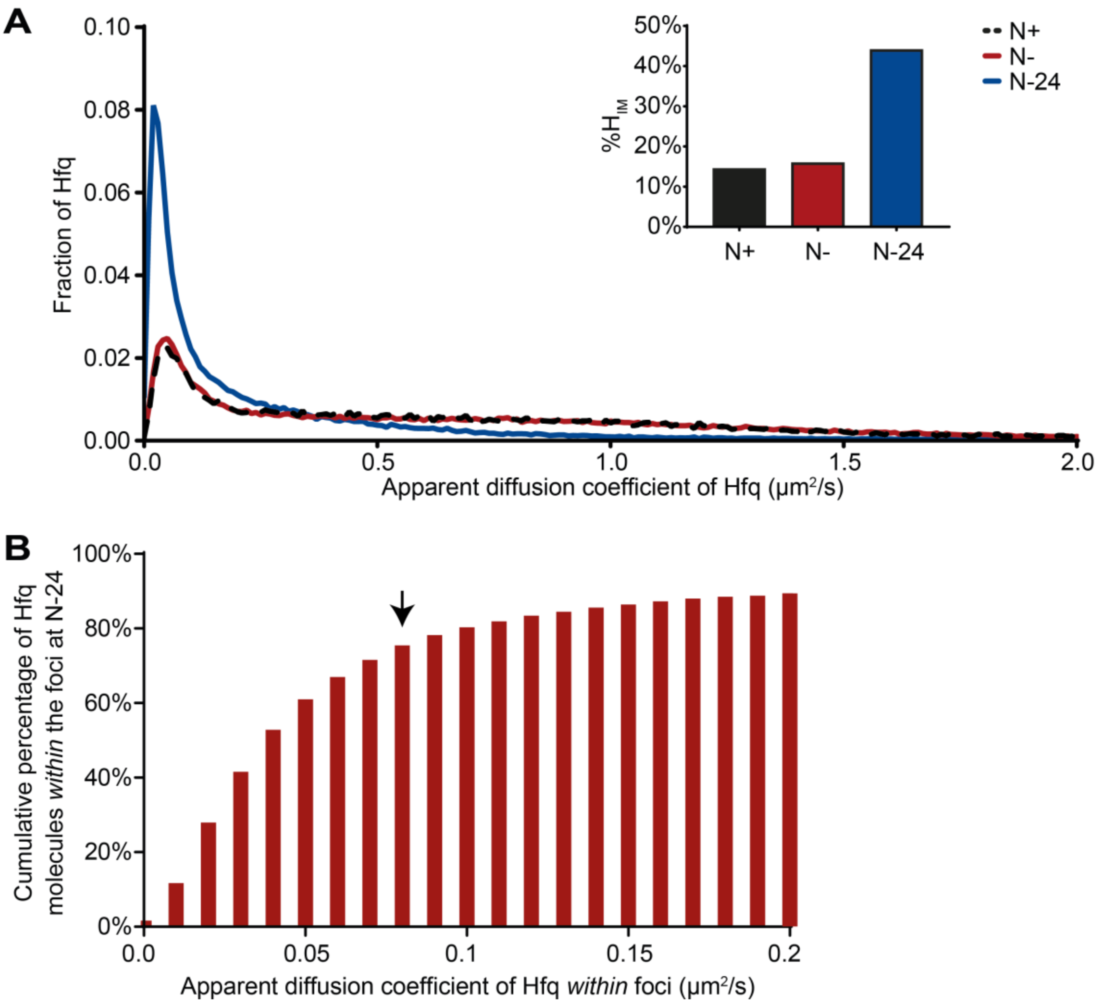

Fig. S3. The majority of Hfq molecules *within* the foci have an apparent diffusion coefficient of <0.08. (*A*) Graphs showing the distribution of the apparent diffusion coefficient of Hfq molecules $\%H_{IM}$ values at N+, N- and N-24 – same data as Figure 3, but with an extended x-axis. (*B*) Graph showing the cumulative proportion of Hfq molecules *within* the foci with increasing D^* values. Arrow indicates cut-off value used for defining $\%H_{IM}$. See text for details.

Figure S4

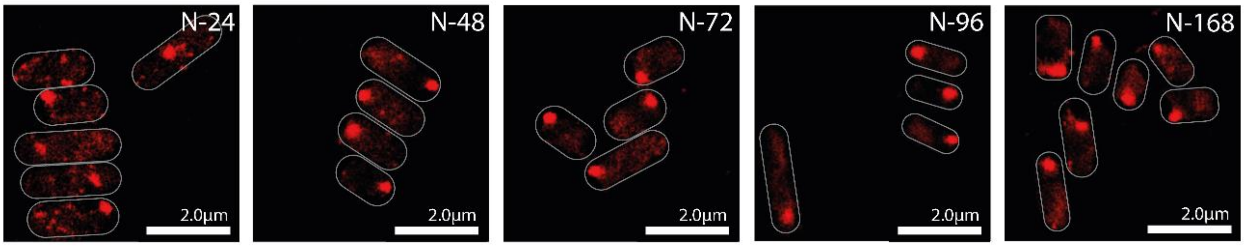

Fig. S4. Hfq foci persist for at least 168 h under N starvation. Representative PALM images of Hfq in *E. coli* cells under long-term N starvation. Images taken at indicated time points.

Figure S5

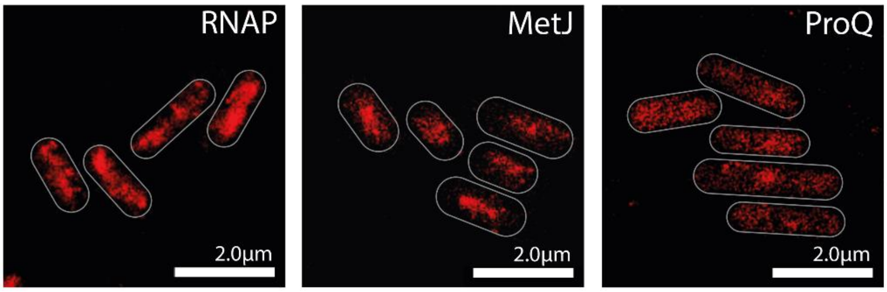

Fig. S5. Foci formation is not a generic feature of proteins during long-term N starvation. Representative PALM images of PAmCherry tagged: RNA Polymerase (RNAP) (*left panel*); MetJ (*middle panel*); and ProQ (*right panel*) in *E. coli* cells at N-24.

Figure S6

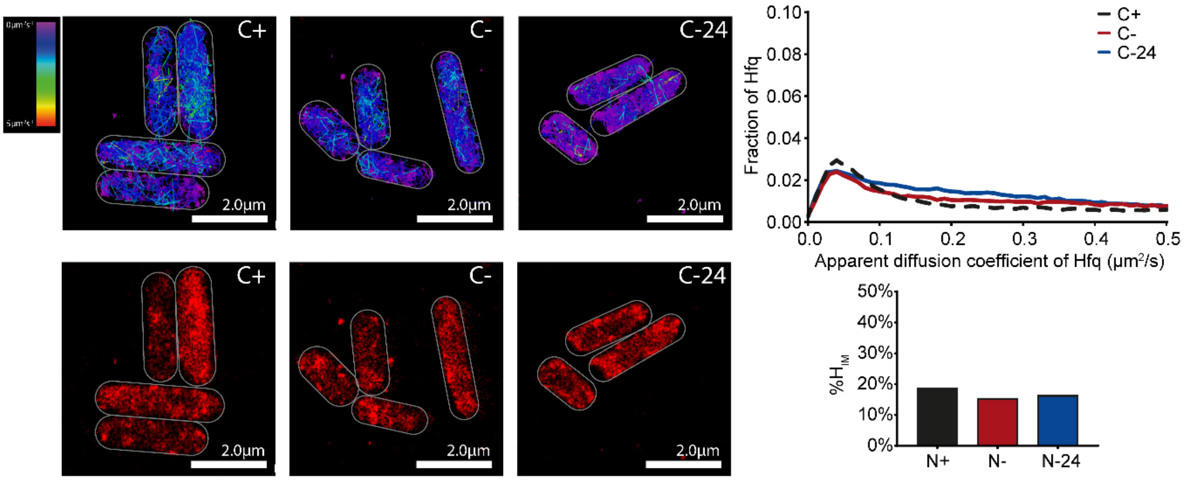

Fig. S6. Hfq does not form foci in long-term C starved *E. coli* cells. Representative single molecule tracks (*top panel*) and PALM images (*bottom panel*) of Hfq in *E. coli* cells from C+, C- and C-24. The graph shows the distribution of apparent diffusion coefficient of Hfq molecules at indicated time points and corresponding %H_{IM} values shown in the inset graph.

Figure S7

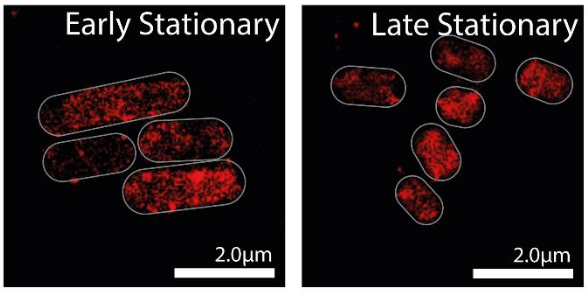

Fig. S7. Hfq does not forms foci during stationary phase in standard lysogeny broth. Representative PALM images of Hfq in *E. coli* cells grown to early (1 h) and late (24 h) stationary phase in standard lysogeny broth.

Figure S8

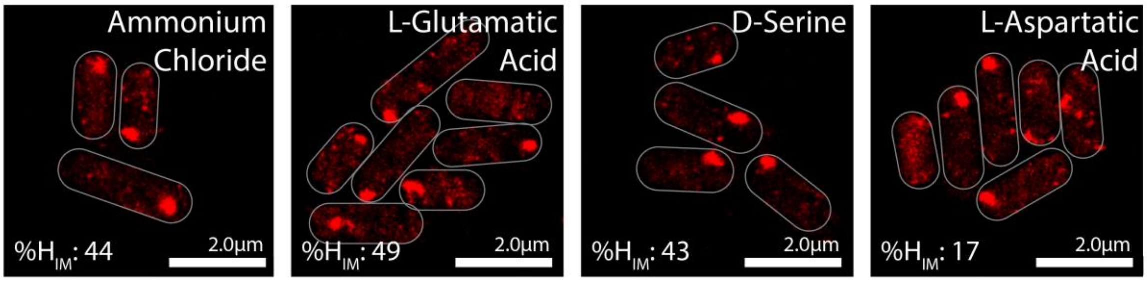

Fig. S8. Hfq foci form in response to starvation of diverse N sources. Representative PALM images of Hfq in *E. coli* cells when 3mM of NH_4Cl (*first panel*), L-Glutamatic acid (*second panel*), D-Serine (*third panel*) or L-Aspartatic acid (*fourth panel*) was used as the sole N source. Cells for imaging were sampled following ~24 h of starvation, using growth in NH_4Cl as the reference time point. The image for ammonium chloride (*first panel*) has been reused from Fig. 3C to allow direct comparison.

Figure S9

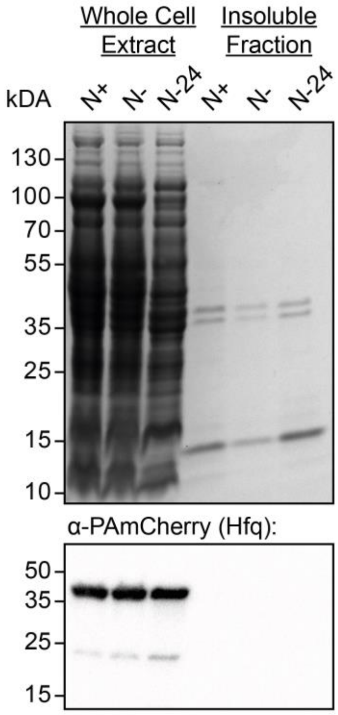

Fig. S9. Hfq foci are not aberrant aggregates of Hfq molecules in long-term N starved *E. coli*. Representative Coomassie stained denaturing gel of whole cell extracts and insoluble protein fraction of *E. coli* cells containing Hfq-PAmCherry from N+, N- and N-24 (*top panel*) and immunoblot of the section containing Hfq (*bottom panel*) using antibodies against the PAmCherry tag.

Figure S10

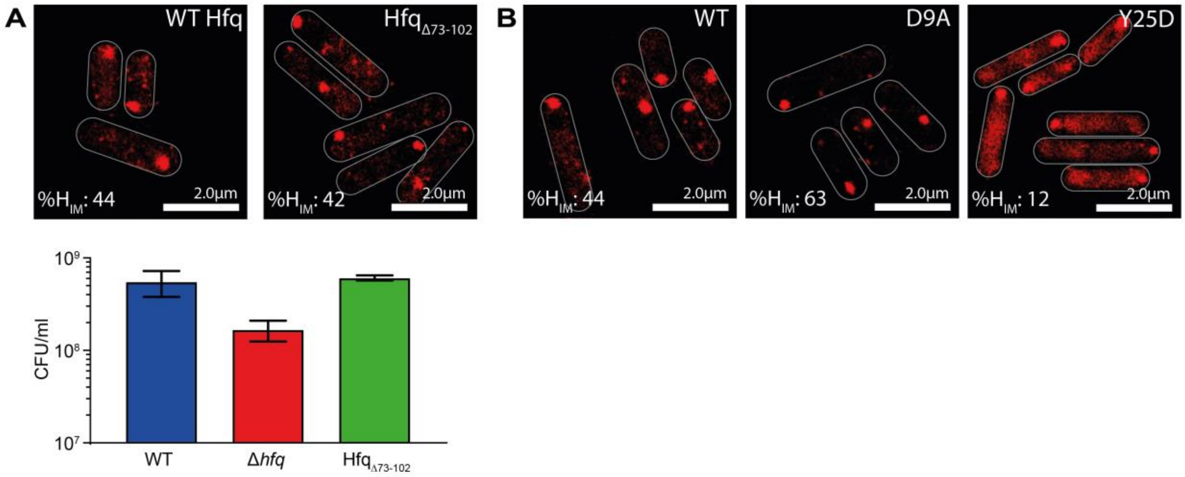

Fig. S10. Hfq foci formation is not dependent on the C-terminal amino acid residues of Hfq, but depends on the RNA-binding activity of Hfq. (*A*) Representative PALM images of full length Hfq (*left panel*) and C-terminally truncated Hfq ($Hfq_{\Delta73\text{-}102}$) (*right panel*) in *E. coli* cells at N-24. The image for WT has been reused from Fig. 3C to allow direct comparison. The graph shows the viability of WT, Δ*hfq* and $Hfq_{\Delta73\text{-}102}$ *E. coli* at N-24 measured by counting CFU. (*B*) Representative PALM images of point mutants of Hfq (WT, D9A and Y25D). Error bars represent standard deviation (n = 3).

Figure S11

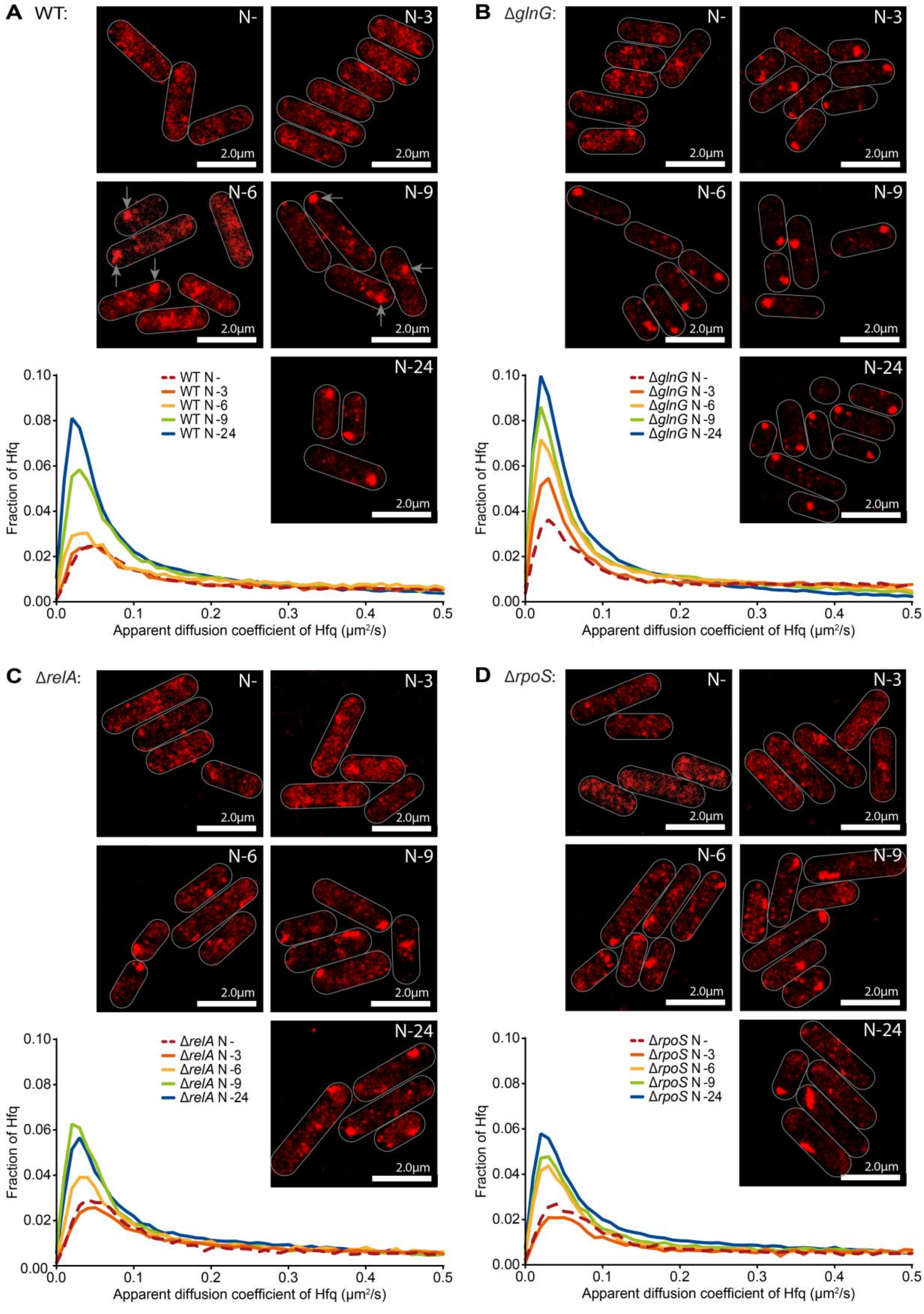

A WT:
N-
N-3
N-6
N-9
N-24
2.0μm
WT N -
WT N -3
WT N -6
WT N -9
WT N -24
Fraction of Hfq
Apparent diffusion coefficient of Hfq (μm²/s)
B ΔglnG:
ΔglnG N -
ΔglnG N -3
ΔglnG N -6
ΔglnG N -9
ΔglnG N -24
C ΔrelA:
ΔrelA N -
ΔrelA N -3
ΔrelA N -6
ΔrelA N -9
ΔrelA N -24
D ΔrpoS:
ΔrpoS N -
ΔrpoS N -3
ΔrpoS N -6
ΔrpoS N -9
ΔrpoS N -24

Fig. S11. Hfq foci formation occurs independently of the NtrC-activated Ntr response in long-term N starved *E. coli*. Representative PALM images of Hfq in *E. coli* cells in (*A*) WT, (*B*) Δ*glnG*, (*C*) Δ*relA* and (*D*) Δ*rpoS* strains, as a function of time under N starvation. In (A), the images for WT have been reused from Fig. 3 (N- and N-24) and Fig. 4A (all other time points) to allow direct comparison. The graphs shows the distribution of apparent diffusion coefficient of Hfq molecules at the indicated sampling time points.

Table S1

E. coli strains and plasmids used in this study

Strains		
Name	Description	Source or Reference
Wild-type (BW25113)	*E. coli* K-12 (*araD-araB*)567 Δ(*rhaD-rhaB*) 568 Δ*lac*Z4787 (*::rrnB-3*) *hsdR*514 *rph*-1	*E. coli* Genetic Stock Center
Δ*hfq* (JW4130)	BW25113 Δ*hfq::kan*	*E. coli* Genetic Stock Center
Δ*rpoS* (JW5437)	BW25113 Δ*rpoS::kan*	*E. coli* Genetic Stock Center
Wild-type (MG1655)	*E. coli* K-12 *rph*-1	*E. coli* Genetic Stock Center
Δ*hfq* (MG1655)	MG1655 Δ*hfq::kan*	
Hfq-PAmCherry	MG1655 *hfq-PAmCherry-kan*	This Study
MetJ-PAmCherry	MG1655 *metJ-PAmCherry-kan*	This Study
RpoC-PAmCherry (KF26)	MG1655 *rpoC-PAmCherry-kan*	[42]
Δ*ProQ*	MG1655 Δ*proQ::kan*	Provided by Prof. Jörg Vogel, University of Wurzburg
Hfq$_{\Delta 73\text{-}102}$ -PAmCherry	MG1655 *hfq*$_{\Delta 73\text{-}102}$*-PAmCherry-kan*	This Study
Hfq(D9A)-PAmCherry	MG1655 *hfq(D9A)-PAmCherry-kan*	This Study
Hfq(Y25D) -PAmCherry	MG1655 *hfq(Y25D)-PAmCherry-kan*	This Study
Δ*glnG* Hfq-PAmCherry	Hfq-PAmCherry Δ*glnG::kan*	This Study
Δ*relA* Hfq-PAmCherry	Hfq-PAmCherry Δ*relA::kan*	This Study
Δ*rpoS* Hfq-PAmCherry	Hfq-PAmCherry Δ*rpoS::kan*	This Study
Plasmids		
Name	Description	Source/Reference
pBAD24-*hfq*-FLAG	pBAD24 expressing *hfq*-3xFLAG under an arabinose inducible promoter	Provided by Prof. Jörg Vogel, University of Wurzburg
pBAD18	Empty pBAD18	[57]
pACYC-*proQ-PAmCherry*	Modified pACYC backbone (-TcR, +MCS) expressing *proQ-PAmCherry* under the native *proQ* promoter	This Study
